# Amikacin dosing in neonates: evaluation of target attainment using a simplified and complex pharmacokinetic model-derived dosing regimen in clinical practice

**DOI:** 10.1128/aac.01118-24

**Published:** 2025-03-11

**Authors:** Marlotte A. A. van der Veer, Anne Smits, Timo R. de Haan, Linda G. W. Franken, Anton H. van Kaam, Caspar J. Hodiamont, Yuma A. Bijleveld, Karel Allegaert, Ron A. A. Mathôt

**Affiliations:** 1 Department of Pharmacy & Clinical Pharmacology, Amsterdam University Medical Center 522567 , Amsterdam, The Netherlands; 2 Neonatal Intensive Care Unit, University Hospitals Leuven 60182 , Leuven, Belgium; 3 Department of Development and Regeneration, KU Leuven 573337 , Leuven, Belgium; 4 Department of Neonatology, Emma Children’s Hospital, Amsterdam University Medical Center 522567 , Amsterdam, The Netherlands; 5 Medical Microbiology, Amsterdam University Medical Center 522567 , Amsterdam, The Netherlands; Providence Portland Medical Center, Portland, Oregon, USA

**Keywords:** amikacin, antimicrobial therapy, neonates, pharmacokinetics, sepsis, target attainment

## Abstract

Amikacin is frequently used for the treatment of neonatal sepsis. The Dutch Pediatric Formulary recommends a complex pharmacokinetic (PK) model-derived dosing regimen, which consists of dosing categories based on postnatal age and weight that results in adequate PK/pharmacodynamic (PK/PD) target attainment. However, a simplified dosing regimen may be easier to apply in clinical practice. We evaluated PK/PD target attainment of amikacin in neonates using this simplified or complex dosing regimen. This retrospective cohort study included neonates with routinely measured amikacin concentrations at the neonatal intensive care units of the Amsterdam University Medical Center (simplified dosing regimen) or University Hospitals Leuven (complex dosing regimen). Peak (C_max_) and trough (C_min_) concentrations and the area under the concentration-time curve (AUC) for the first dosing interval were calculated by Bayesian estimation for both populations. Targets of C_max_ (≥15, ≥25, and ≥35 mg/L), C_min_ (≤3 and ≤5 mg/L), and AUC/minimal inhibitory concentration (MIC: 2, 4, and 8 mg/L for *Enterobacterales* species) for bacteriostasis and 1-log reduction were evaluated. A target attainment of ≥90% was considered adequate. In total, 366 neonates (768 concentrations) and 579 neonates (1,195 concentrations) received the simplified and complex dosing regimen, respectively. Both regimens achieved target attainment of 100% for C_max_ ≥ 15 mg/L, C_min_ ≤ 5 mg/L, AUC/MIC for bacteriostasis, and AUC/MIC for 1-log reduction up to a MIC of 2 mg/L. Target attainment was achieved for less stringent targets (C_max_ ≥ 15 mg/L, C_min_ ≤ 5 mg/L, and AUC/MIC for bacteriostasis) with the simplified and complex amikacin dosing regimen. Clinicians can choose one of both dosing regimens, depending on their local circumstances, and the availability of integrated (electronic) prescription tools.

## INTRODUCTION

The aminoglycoside amikacin has a prominent place in the treatment of neonatal sepsis due to its activity against gram-negative bacteria, as well as its action on staphylococci (1). Precision dosing of amikacin in neonates is challenged by a high interindividual variability (IIV) in its pharmacokinetics (PK), resulting from differences in ongoing maturation of renal function, body composition, and even pathophysiological states such as sepsis (2–4). Several population PK studies have sought to identify patient-specific predictors that determine IIV and to propose PK model-derived dosing regimens for neonates to achieve efficacy targets while minimizing the risk for unwanted side effects (2, 3, 5–7).

A commonly used amikacin dosing regimen has been derived from the amikacin PK model of De Cock et al., in which 10 different dosing categories have been proposed dependent on both postnatal age (PNA) and body weight (2). The dose is further adapted in case of ibuprofen co-administration due to its effect on the renal clearance of amikacin. This dosing regimen has been adopted by the Dutch Pediatric Formulary (8). While this complex dosing regimen has proven to suffice in reaching adequate peak (C_max_) and trough (C_min_) concentrations in a prospective evaluation study from Smits et al. (9), it is challenging to apply in clinical practice, especially if no electronic prescription algorithm is used (Table 1). Furthermore, the predictive performance of this PK model has not yet been externally validated in a neonatal population of a different center. At the Amsterdam Medical University Center (Amsterdam UMC), a simplified dosing regimen with two dosing categories, roughly based on the Dutch Pediatric Formulary, has been adopted (see Table 2). Therapeutic drug monitoring (TDM) is routinely performed after the first dose of this simplified dosing regimen, and in our experience, adequate amikacin plasma concentrations are also attained immediately after the first dose.

**TABLE 1:** Complex model-derived dosing regimen of amikacin in neonates based on postnatal age and current body weight; in case of ibuprofen co-administration, the dosing interval is prolonged with 10 hours (2, 9)

Postnatal age (days)	Group	Current weight (grams)	Amikacin dose
<14	1	<800	16 mg/kg every 48 hours
	3	800 to 1,200	16 mg/kg every 42 hours
	5	1,200 to 2,000	15 mg/kg every 36 hours
	7^*a*^	2,000 to 2,800	15 mg/kg every 30 hours
	9^*a*^	>2,800	15 mg/kg every 24 hours
≥14	2	<800	20 mg/kg every 42 hours
	4	800 to 1,200	20 mg/kg every 36 hours
	6	1,200 to 2,000	18 mg/kg every 30 hours
	8	2,000 to 2,800	18 mg/kg every 24 hours
	10	>2,800	18 mg/kg every 20 hours

^*a*^ An additional prolonged dosing interval of +6 hours is suggested for subgroups 7 and 9 in the original publication (9). Since amikacin plasma concentrations were measured without this extended interval, the dosing regimen as listed in this table is used for the current evaluation.

**TABLE 2:** Simplified dosing regimen of amikacin in neonates based on gestational age and current body weight

Gestational age (weeks)	Postnatal age (days)	Amikacin dose
<30 weeks	≤30	15 mg/kg every 36 hours
≥30 weeks	≤30	15 mg/kg every 24 hours

Interestingly, there is no consensus about the optimal PK/pharmacodynamic (PD) target for amikacin (1). In clinical context, the C_max_/minimum inhibitory concentration (MIC) ratio is typically used as a PK/PD index for aminoglycoside efficacy, while C_min_ is generally related to toxicity. However, more recently, the use of the area under the concentration-time curve (AUC)/MIC ratio has also been resuggested as efficacy target (10, 11).

In the current study, we evaluated the predictive performance of the previously published PK model of De Cock et al. for neonates receiving amikacin in an independent clinical cohort (2). Second, we evaluated target attainment of amikacin when using either this simplified or complex model-derived dosing regimen for different PK/PD targets in two cohorts of neonates admitted to two neonatal intensive care units (NICU) in the Netherlands and Belgium.

## RESULTS

### Patients

In total, 768 amikacin plasma concentrations from 366 neonates receiving the simplified dosing regimen were available, while 1,195 concentrations from 579 neonates receiving the complex dosing regimen were available as previously described (9). The patient characteristics of neonates included in both data sets are shown in Table 3. Additionally, Tables S1 and S2; Supplementary Data 2 displays the patient count per dosing category for the simplified and complex dosing regimen. Overall, more premature neonates received the simplified dosing regimen (median postmenstrual age [PMA] 30 [range 24–45] weeks vs. PMA 34 [range 24–54] weeks), also reflecting a significantly lower median birth weight (1,100 g vs. 2,150 g). Also, as amikacin is mostly used for the treatment of late onset sepsis in the Amsterdam UMC, but also for early onset sepsis in the University Hospitals Leuven (UHL), median PNA at the time of amikacin treatment was significantly higher in the data set of neonates receiving the simplified dosing regimen (9 vs. 2 days, *P* < 0.01). Serum creatinine levels were significantly higher in neonates receiving the complex dosing regimen (88 vs. 42 μmol/L, *P* < 0.01), likely due to the influence of maternal serum creatinine levels during the initial days of life and renal maturational differences (12). The most frequently isolated pathogens in positive blood cultures were *Escherichia coli* and *Klebsiella oxytoca* for neonates in Amsterdam (own unpublished clinical data) and *Escherichia coli* and *Staphylococcus epidermidis* for those in Leuven (9).

**TABLE 3:** Characteristics of neonates receiving the simplified dosing regimen in the Amsterdam UMC and the complex dosing regimen in University Hospitals Leuven (9)^*e*^

Characteristic^*a*^	Simplified dosing regimen (*n* = 366)	Complex dosing regimen (*n* = 579)	*P* value
Male, *n* (%)	216 (59%)	324 (56%)	0.62
GA, weeks^*b*^	28.7 (24.0–42.1)	34 (24–41)	<0.01
GA < 30 weeks, *n* (%)	209 (56.9%)	91 (15.7%)	
GA ≥ 30 weeks, *n* (%)	158 (43.1%)	489 (84.3%)	
GA ≥ 37 weeks, *n* (%)	46 (12.5%)	212 (36.6%)	
PNA (days)^*c*^	9 (0–31)	2 (1–30)	<0.01
PMA (weeks)^*c*^	30.2 (24.6–44.6)	34 (24–54)	<0.01
Birth weight (g)^*b*^	1,100 (440–5,200)	2,150 (420–4,850)	<0.01
Current weight (g)^*c*^	1,100 (455–5,500)	2,120 (420–5,040)	<0.01
SCr (μmol/L)^*c*^^,*d*^	42 (11–194)	88 (11–316)	<0.01
Co-administration of ibuprofen, *n* (%)^*c*^	7 (2%)	29 (5%)	<0.01
Total number of samples	768	1,195	
C_min_ samples, *n* (%)	242 (32%)	741 (62%)	
C_max_ samples, *n* (%)	402 (52%)	417 (35%)	
BLQ samples, *n* (%)	61 (7.9%)	52 (4.4%)	

^*a*^ Baseline characteristics are depicted by median and range for continuous variables and percentages for categorical variables.

^*b*^ Measured at birth.

^*c*^ Measured around amikacin administration.

^*d*^ Creatinine measured in *n* = 302 neonates (simplified dosing regimen) and *n* = 571 neonates (complex dosing regimen).

^*e*^ GA, gestational age; BLQ, below limit of quantification; C_max_, peak concentration (defined as time after amikacin dose < 2 hours); C_min_, trough concentration (defined as time after amikacin dose > 20 hours); *n*, number; PMA, postmenstrual age; PK, pharmacokinetic; PNA, postnatal age; SCr, serum creatinine.

### External validation of the population PK model

The external validation of the population PK model of the study by De Cock et al. was conducted using all data from neonates receiving the simplified dosing regimen (2). The results are shown in Supplementary Data 3. Individual- and population-predicted concentrations plotted against observed concentrations demonstrate a uniform distribution of data points around the unity line (Fig. S1). This indicates an even dispersion of IIV across the neonatal population, a trend further supported by plots depicting IIV in clearance against PNA, birth weight, and current weight (Fig. S2). Notably, no discernible trend is evident in normalized prediction distribution error (NPDE) plotted against time or predicted concentrations, suggesting model accuracy. However, the mean of the NPDE distribution significantly deviates from 0, indicating a non-normal distribution (Fig. S3). In contrast, the visual predictive checks (VPC) effectively captured the data (Fig. S4). Re-estimation of the final PK parameters using the data set with neonates receiving the simplified dosing regimen yielded values largely consistent (≤20% difference), although there was a reduced covariate effect of PNA on clearance and an increased IIV on clearance (Table S3). Bias and imprecision were low for the C_min_ but were considerably higher for the C_max_, as evidenced by a mean prediction error (MPE) of −2.1 mg/L (−2.9 to −1.7 mg/L) and a root mean squared error (RMSE) of 6.3 mg/L (95% CI: 0.5 to 12.0 mg/L) (Table S4). As C_max_ values are predominantly influenced by the central volume of distribution (V1), the absence of IIV on this parameter might explain the model’s challenges in accurately predicting the C_max_.

### Development of an optimized population PK model

To create an optimized population PK model with unbiased C_max_ estimations, both data sets (neonates receiving the simplified and complex dosing regimen) were combined to form a model-building data set (containing 75% of neonates, *n* = 710 with 1,412 amikacin samples) and a validation data set (containing 25% of neonates, *n* = 367 with 768 amikacin samples) at random. The final PK parameters from De Cock et al. were re-estimated using the model-building data set (2). The IIV on V1 could also not be estimated and was therefore fixed at a biologically plausible value. A value of 30% was selected for IIV for V1, considering the range of IIVs found in previously published neonatal amikacin PK models (0% to 30%) and its consistency with variability observed in neonatal gentamicin PK models (another aminoglycoside with similar PK properties) (3, 6, 7, 13). Sensitivity analyses were conducted to validate the appropriateness of a 30% IIV on V1 for our data. Results are presented in Supplementary Data 4.

### Evaluation of target attainment

**Fig 1:**
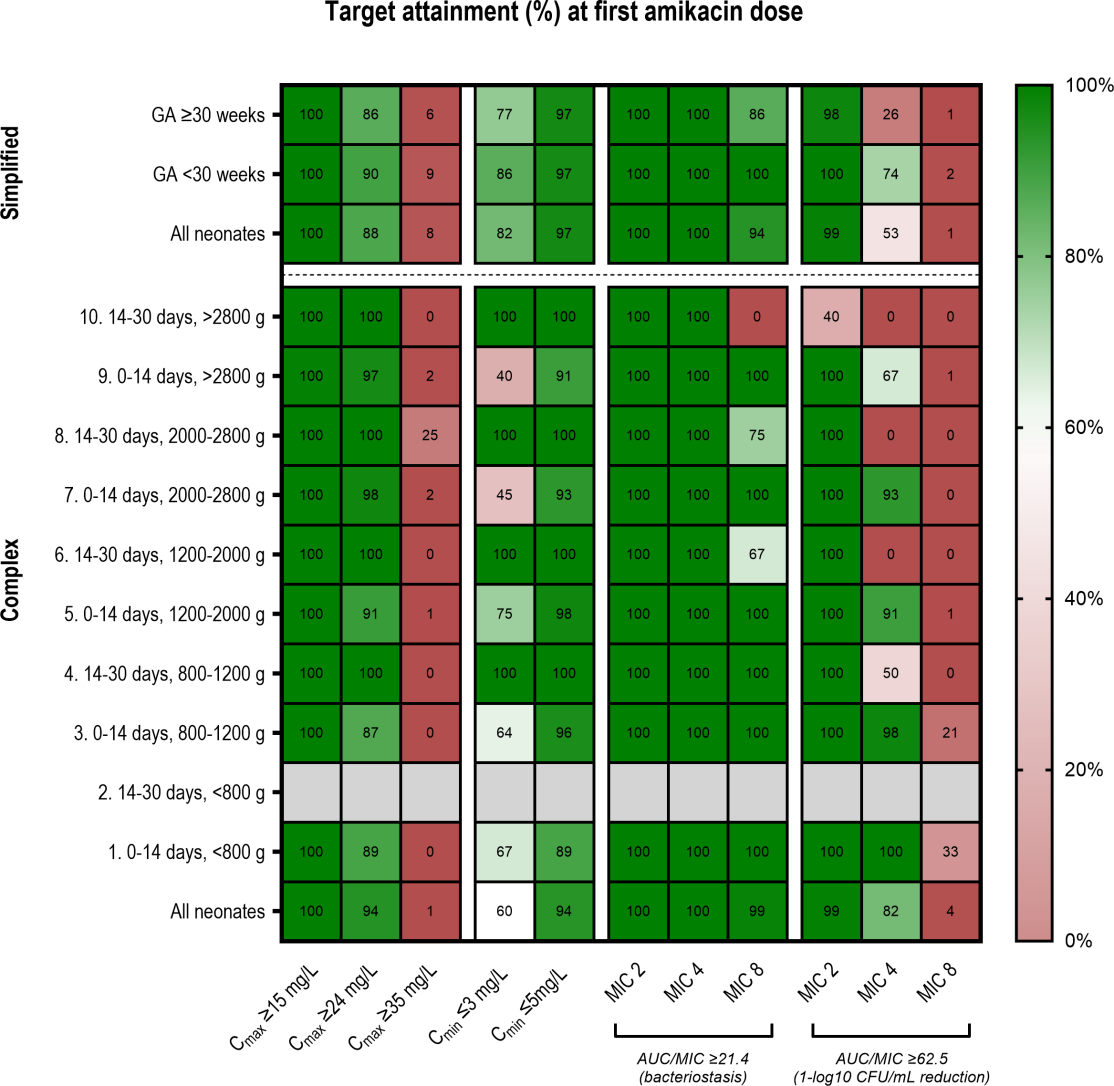
Target attainment of amikacin for the first dose for neonates receiving the simplified dosing regimen or the complex dosing regimen per dosing category and in total. Target attainment is calculated for different targets for efficacy and toxicity, including peak concentrations (C_max_), area under the curve over minimum inhibitory concentration (AUC/MIC) for bacteriostasis or reduction, and trough concentrations (C_min_). The optimized pharmacokinetic model as described was used to calculate precise C_min_, C_max_, and AUC values at the first dose.

Precise amikacin C_max_, C_min_, and AUC values for both data sets (neonates receiving the simplified and complex dosing regimen) were determined using the optimized PK model. Figure 1 illustrates the percentage of patients achieving a PK/PD target per dosing category for either the simplified or complex regimen. Overall, among neonates receiving the simplified dosing regimen, 82% had a C_min_ ≤ 3 mg/L and 97% had a C_min_ ≤ 5 mg/L, whereas 60% and 94% of neonates receiving the complex dosing regimen reached these targets, respectively. For C_max_ values, 88% of neonates reached the target ≥ 24 mg/L with the simplified dosing regimen and 94% of neonates with the complex dosing regimen. All neonates in both data sets reached C_max_ ≥ 15 mg/L. Neonates reaching C_max_ ≥ 35 mg/L were limited to 8% and 1% within the simplified and complex dosing regimens, respectively, indicating that overexposure is rare and reflecting the distribution characteristics for aminoglycosides in this population. Optimal target attainment (≥90%) was achieved for neonates using the simplified and complex dosing regimen with target values of C_min_ ≤ 5 mg/L and C_max_ ≥ 15 mg/L, which are the targets used in clinical practice at Amsterdam UMC. Target attainments for the targets used in Leuven (C_min_ ≤ 3 mg/L and C_max_ ≥24 mg/L) were 82% and 88% for the simplified dosing regimen, respectively, while for the complex dosing regimen, those were 60% and 94%, respectively. No apparent differences were observed among the various dosing categories for either the simplified or complex dosing regimens.

The AUC/MIC targets for achieving bacteriostasis across the entire wild-type MIC distribution (≤8 mg/L) of *Escherichia coli* were attained in ≥90% of neonates in both dosing groups. Target attainment was achieved with either dosing regimen when aiming for 1-log 10 CFU/mL reduction when considering a MIC ≤ 2 mg/L. The target attainment per subgroup of the two different dosing regimens is shown in Supplementary Data 5.

## DISCUSSION

To the best of our knowledge, this is the first study to utilize clinical data from a large number of neonates from two centers to assess whether a simplified dosing regimen and a complex amikacin model-derived dosing regimen effectively achieve the conventionally used target amikacin concentrations (C_min_, C_max_) as set in clinical practice. Additionally, we evaluated whether commonly used dosing regimens in neonatal populations reached AUC/MIC targets for the first dosing interval when targeting *Enterobacterales* species.

The discrepancy in target values used in clinical practice between the Amsterdam UMC and UHL underscores the challenge in establishing the optimal PK/PD parameter for amikacin. Traditionally, a C_max_-to-MIC ratio of at least 8 to 10 has been used as target for aminoglycosides, as it has been shown to correlate with improved survival in adult patients (14). For instance, a C_max_ of 24 mg/L is assumed to correspond to a MIC of 3 mg/L for *Escherichia coli*, and while this may suffice for some isolates, it falls short for the epidemiological cut-off (ECOFF) set at 8 mg/L for *Escherichia coli* (15). However, achieving a C_max_ of at least 64 mg/L would prove impractical if a sufficiently low C_min_ is required to prevent toxicity. Consequently, The European Committee on Antimicrobial Susceptibility Testing (EUCAST) suggests administering amikacin as part of combination therapy (with another active antimicrobial agent) as these may exhibit synergistic effects. However, it remains uncertain whether lower PK/PD targets can be utilized in the presence of synergy during combination therapy (11). As part of the concentration-dependent bactericidal action of aminoglycosides, a postantibiotic effect has been observed, wherein bacterial eradication persists even after concentrations fall below the MIC, allowing for longer dosing intervals of aminoglycosides while still maintaining efficacy and reducing toxicity (16, 17). A relationship between the AUC/MIC ratio and clinical efficacy has been shown for aminoglycosides, primarily derived from clinical data on tobramycin use in cystic fibrosis patients (18–20). More recently, recommendations from EUCAST (2024) and The United States Committee on Antimicrobial Susceptibility Testing (USCAST) (21) have shifted to using the AUC/MIC ratio for amikacin, a concept which has not been validated in neonates but is increasingly used as PK/PD target in neonatal studies (10, 11, 21).

Employing the AUC/MIC ratio as a PK/PD target necessitates the use of an appropriate amikacin population PK model tailored to this specific population. Also, given the high IIV of amikacin (mainly in clearance), the use of a PK model to optimize dosing immediately following the first dose is imperative (19). Ideally, a population PK model is externally validated using independent data to evaluate its predictive performance (22). In our study, we externally validated an amikacin PK model using an independent data set from clinical practice (2). We found that the amikacin population PK model of De Cock et al. was generally well suited for Dutch neonates, except for the estimation of C_max_ values (2). Since we still could not estimate IIV on V1 in a combined data set, we fixed IIV on V1 at a biologically plausible level (30%) and confirmed this assumption through sensitivity analyses as a proof of concept. Estimating the IIV on V1 for amikacin appears challenging in neonates, as either no estimation could be made or the estimation exhibited high shrinkage, resulting in less accurate posterior predictions (2, 6, 23). One recently published amikacin PK model in neonates did succeed in estimating IIV on V1 but appeared less suitable for our population, which is primarily composed of premature neonates, as the model was developed using data exclusively from term neonates (3).

In this study, we showed that most PK/PD targets are met when using a simplified dosing regimen. The use of a simplified dosing regimen may reduce the occurrence of prescription and medication administration errors compared with a more intricate regimen, especially when the latter is not implemented correctly. Although the occurrence of medication errors was not the focus of this current study, they are frequently observed in NICUs due to various factors, underlining the importance of seeking ways to enhance medication safety for neonates (24). The complex model-derived amikacin dosing regimen is integrated in the electronic prescription system in the UHL, using pre-programmed templates. This supports feasibility of integrating complex dosing regimens in clinical care.

Our study has limitations to address. First, due to its retrospective nature, not all variables influencing amikacin PK could be collected from the electronic medical records. For example, recent studies in neonates revealed that amikacin clearance was affected by pathophysiological conditions as clinical shock and sepsis, but also by perinatal asphyxia treated with therapeutic hypothermia (3, 25). Also, errors in medication administration and timing of amikacin sample collection cannot be entirely ruled out. Moreover, the retrospective design of our study did not allow us to include comparable neonates in terms of clinical characteristics and amikacin treatment indications. These differences would confound the outcomes if we were to conduct a non-inferiority analysis, as non-inferiority studies should be conducted within a trial context with largely comparable groups to minimize the risk of bias (26). Hence, we opted to present the percentages of target attainment for the two different dosing regimens, enabling clinicians to exercise individual clinical judgment based on their own specific circumstances. Assessing raw data from a large number of neonates across two different clinical settings has not been previously attempted. Previous retrospective comparisons of different dosing regimens have been conducted within single centers, without considering multiple PK/PD targets (27, 28).

Second, our study did not examine clinical outcomes and was not powered to relate C_max_ or AUC/MIC ratio to the efficacy of the amikacin therapy. Nevertheless, our findings provide a good insight into the achievement of various PK/PD targets with different amikacin dosing regimens currently used in clinical practice. Further research should be conducted to establish the link between a PK/PD target and clinical outcomes in neonates.

### Conclusion

In conclusion, this is the first study in which retrospectively collected data from neonates from two centers are used to evaluate PK/PD target attainment of a complex amikacin model-derived dosing regimen and a simplified dosing regimen. Conventional targets, such as C_max_ ≥ 15 mg/L and C_min_ ≤ 5 mg/L, were successfully reached in ≥90% of patients with either the simplified or complex dosing regimen. Clinicians can choose one of both dosing regimens, depending on their local circumstances, and the availability of integrated (electronic) prescription tools. However, AUC/MIC targets (MIC > 2 mg/L) for a 1-log 10 CFU/mL reduction across the entire wild-type MIC distribution of *Escherichia coli* were not met with either regimen. Bacteriostasis targets were reached, which may be acceptable if amikacin is used as part of combination therapy. Further research is needed to determine the optimal PK/PD target for treating neonatal sepsis when amikacin is used in combination therapy. Furthermore, we assessed the predictive performance of a previously published amikacin population PK model using an independent data set of neonates and optimized this model to allow for a more accurate prediction of C_max_.

## MATERIALS AND METHODS

### Study design

This retrospective cohort study was carried out within the NICUs of the Amsterdam UMC in the Netherlands and the University Hospitals Leuven (UHL) in Belgium. The objective was to evaluate the proportion of neonates achieving amikacin targets with either the simplified or complex dosing regimen for amikacin.

Two data sets were used for this assessment: (i) a retrospectively collected data set containing neonates receiving the simplified amikacin dosing regimen in the Amsterdam UMC between January 2018 and December 2024 and (ii) a published data set containing neonates receiving the complex amikacin dosing regimen in the UHL between July 2011 and December 2012 (9). All raw data from this study were available for further analysis. The study was approved by the ethical boards of the Amsterdam UMC and UHL.

### Study population

All neonates admitted to the two NICUs in the aforementioned time period with amikacin plasma samples available were considered for inclusion. Exclusion criteria were a PNA of >30 days, use of a dosing regimen different from the two under evaluation, or missing patient data. Clinical characteristics and prescription data were obtained from electronic medical records and encompassed gestational age, PNA, sex, serum creatinine, birth weight, current body weight, amikacin dose, or concurrent ibuprofen use at time of amikacin administration. Current body weight, birth body weight, and concurrent ibuprofen use were incorporated as covariates on amikacin clearance in the PK model of De Cock et al. (2). More study details for the complex dosing regimen can be found in the original publication (9).

Patient characteristics were compared between the two data sets using Pearson’s chi-square test or the Mann-Whitney U test. *P* values < 0.05 were considered statistically significant. Data were analyzed using IBM SPSS Statistics Version 28.0.

### Dosing and blood sampling

Amikacin was administered by an intravenous infusion over 20 to 30 minutes. Neonates admitted to the NICU at Amsterdam UMC (simplified dosing regimen) received amikacin based on the dosing regimen detailed in Table 2, whereas in neonates admitted to the NICU at UHL (complex dosing regimen), amikacin was administered following the dosing regimen outlined in Table 1.

Samples for TDM were collected as part of routine clinical care just prior to the second amikacin dose (C_min_) in both NICUs and 1 hour after administration (C_max_) of the first dose (Amsterdam UMC) or the second dose (UHL). Subsequent sampling occasions were based on the discretion of the physician and pharmacist. TDM data for the simplified dosing regimen were collected retrospectively from model-informed precision dosing software MW/Pharm and the InsightRX Nova precision dosing platform.

### Drug assay

All amikacin plasma samples from the complex dosing regimen were measured using a fluorescence polarization immunoassay (TDx, Abbott) or kinetic interaction of micro-particles in solution (KIMS) immunoassay (Cobas c, Roche/Hitachi) as described in more detail elsewhere (9). Similarly, amikacin concentrations in the simplified dosing regimen were analyzed using the KIMS immunoassay (Cobas c, Roche/Hitachi). Precision values for all three assays were <5%, with the lower limit of quantification set at 0.8 mg/L.

### Clinical practice evaluation

#### External validation of population PK model

The complex dosing regimen (Table 1) has been derived from the population PK model of De Cock et al. (2). Population PK parameter estimates from this model are shown in Table S3.

As Bayesian estimates were calculated for C_max_, C_min_, and AUC for both study populations, it was necessary to assess the suitability of the PK model to estimate these values. Therefore, the predictive performance of the PK model from De Cock et al. was evaluated with the neonates receiving the simplified dosing regimen (external validation) (2). Suitability of this PK model for neonates receiving the complex dosing regimen was already assessed in the original publication of Smits et al. (9). For this current external validation, several tests such as visual inspection of goodness-of-fit plots, calculation of bias and imprecision, VPC, and NPDE analysis were performed as described in the Supplementary Data 1. Samples from multiple dosing occasions were included in this external validation.

#### Evaluation of target attainment

Target attainment for amikacin was assessed for the simplified and complex dosing regimen for the first dosing interval. Typically, amikacin PK/PD studies aim for a probability of target attainment in ≥90% of patients (29–31), although a lower target attainment percentage of ≥80% is also described (32). In this current study, a target attainment of ≥90% was considered adequate.

Different therapeutic targets were evaluated: (i) C_max_ ≥ 24 mg/L (9); (ii) C_max_ ≥ 15 mg/L (33); (iii) C_max_ ≥ 35 mg/L (to assess overexposure) (9); (iv) AUC/MIC of ≥21.4 for bacteriostasis of *Enterobacterales* in a murine model, which is considered a reasonable endpoint for complicated urinary tract infections and complicated intra-abdominal infections; and (v) AUC/MIC of ≥62.5 for 1−log_10_ CFU/mL reduction of *Enterobacterales* in a murine model*,* which is considered a reasonable endpoint for hospital-acquired or ventilator-associated pneumonia (11, 21). While these targets are not validated in neonates, they represent the only available AUC/MIC targets for *Enterobacterales* (21). Targets for AUC/MIC were determined using amikacin MIC values for *Escherichia coli* as reported by EUCAST of prevalent MIC of 2 and 4 mg/L as well as using the ECOFF of 8 mg/L to evaluate the coverage of the whole wild-type distribution (15). For nephro- and ototoxicity purposes, amikacin C_min_ of (i) ≤3 mg/L and (ii) ≤5 mg/L were considered (9, 34). Precise C_max_ values were defined as concentrations calculated 1 hour after the start of the infusion of the first dose and C_min_ values as concentrations calculated just before the second dose, and AUC was calculated over the given dosing interval. The validated amikacin PK model was used to calculate these concentrations. All targets were evaluated for the first dosing interval to assess early target attainment.

## Data Availability

All data are available upon reasonable request.

## SUPPLEMENTAL MATERIAL

Supplemental material aac.01118-24-s0001.docx Supplemental methods, tables, and figures.

ASM does not own the copyrights to Supplemental Material that may be linked to, or accessed through, an article. The authors have granted ASM a non-exclusive, world-wide license to publish the Supplemental Material files. Please contact the corresponding author directly for reuse.
