# Supplementary material for: Amikacin dosing in neonates: evaluation of target attainment using a simplified and complex pharmacokinetic model-derived dosing regimen in clinical practice
Source: Antimicrob Agents Chemother. 2025 Mar 11;69(4):e01118-24. doi: 10.1128/aac.01118-24 (PMC11963607; doi:10.1128/aac.01118-24)
Supplement: Supplemental material — Supplemental methods, tables, and figures. [file aac.01118-24-s0001.docx]

**Supplementary Data
Supplementary data 1: Method for external validation of amikacin population pharmacokinetic (PK) model.**

*External validation of population PK model*The external validation was conducted using the PK model of De Cock et al. and data collected in neonates receiving the simplified dosing regimen (1). Final population PK parameter estimates for the PK model of De Cock et al. are shown in table S3. This two-compartment model was based on 2186 observations of 877 neonates and equalized central volume of distribution (V1) to peripheral volume of distribution (V2), and estimated intercompartmental clearance (Q) as a fraction of clearance (CL).The non-linear mixed-effects modeling (NONMEM) software package was used. Population-predicted concentrations were calculated using NONMEM by fixing the final parameters of the original model using the MAXEVAL = 0 command. These predicted concentrations were then graphically compared to the observed concentrations across all concentrations, as well as stratified into C_min_ and C_max_ values. Visual predictive checks (VPC) and normalized prediction distribution error (NPDE) analyses were conducted, both with n=1000 simulations (2, 3). Also, parameters of the PK model were re-estimated using data from neonates receiving the simplified dosing regimen in which the re-estimated values should ideally be within ±20% of the original estimates (4). Finally, bias and imprecision were calculated for all amikacin concentrations and for C_min_ and C_max_ values separately as any bias or imprecision would have more significant implications for low amikacin concentrations compared to high concentrations. The following equations were used (5):

$$Prediction error \left( PE \right)=model predicted concentration-measured concentration$$

$$\boldsymbol{Bias:} Mean prediction error \left( MPE \right)= \frac{\sum_{j = 1}^{N} \left( {PE}_{j} \right)}{N}$$

$$\boldsymbol{Imprecision:}Root mean squared error \left( RMSE \right)= \sqrt{\frac{\sum_{j = 1}^{N} {({PE}_{j})}^{2}}{N}}$$

In this context, C_max_ values were defined as time after amikacin dose <2 hours, while C_min_ values were defined as time after amikacin dose >20 hours. If unsatisfactory results were obtained from any of the external validation tests, alternative approaches were explored. These included a review of the literature for other suitable neonatal amikacin PK models or development of an adjusted amikacin population PK model by merging the two datasets into a model-building dataset (75% of total neonates combined) and external dataset (25% of total neonates combined).

**Supplementary data 2: patient count per dosing category**

| **Postnatal age (days)** | **Group** | **Current weight (grams)** | **Simplified dosing regimen (n)** | **Complex dosing regimen (n)** |
| --- | --- | --- | --- | --- |
| <14 | 1 | < 800 | 63 | 32 |
|  | 3 | 800 to 1200 | 85 | 61 |
|  | 5 | 1200 to 2000 | 70 | 178 |
|  | 7 | 2000 to 2800 | 27 | 144 |
|  | 9 | > 2800 | 41 | 210 |
| ≥14 | 2 | < 800 | 11 | 0 |
|  | 4 | 800 to 1200 | 38 | 6 |
|  | 6 | 1200 to 2000 | 15 | 7 |
|  | 8 | 2000 to 2800 | 9 | 9 |
|  | 10 | > 2800 | 7 | 10 |

**Table S1.** Number of neonates per dosing category for the complex dosing regimen across both the dataset including neonates receiving the simplified dosing regimen or complex dosing regimen, respectively, illustrating the distribution of neonates among the different dosing subgroups (6).

| **Gestational age (weeks)** | **Group** | **Simplified dosing regimen (n)** | **Complex dosing regimen (n)** |
| --- | --- | --- | --- |
| <30 | 1 | 209 | 91 |
| ≥30 | 2 | 158 | 489 |

**Table S2.** Number of neonates per dosing category for the complex dosing regimen across both the dataset including neonates receiving the simplified dosing regimen or complex dosing regimen, respectively, illustrating the distribution of neonates among the different dosing subgroups (6).

**Supplementary data 3: Results of external validation of amikacin population PK model**

**
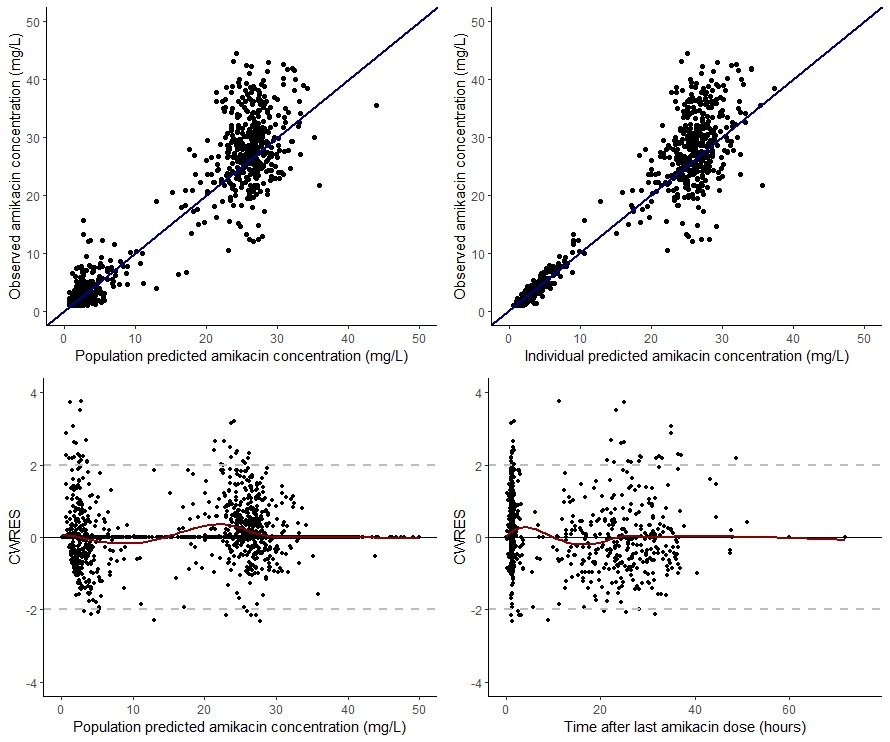
Goodness-of-fit plot (external validation)**

**Figure S1:** Goodness-of-fit plot of the pharmacokinetic model of De Cock et al. using data from neonates receiving the simplified dosing regimen (1). The blue line represents the line of unity and the red line represents the correlation of observations and predictions. The model appears to predict C_max_ values less accurately compared to C_min_ values, which becomes apparent when comparing individual predictions against observed concentrations (right upper figure). C_max_, peak concentrations; C_min_, trough concentrations; CWRES, conditional weighted residuals.

**Interindividual variability on amikacin clearance versus covariates**


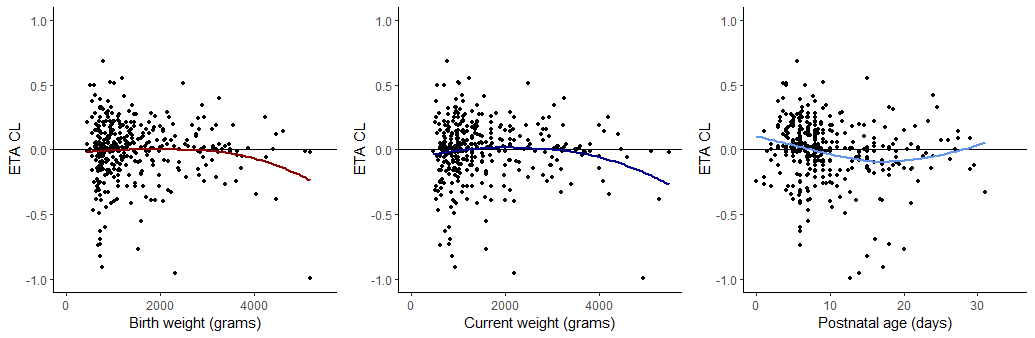


**Figure S2:** Interindividual variability (ETA) on clearance (CL) versus birth body weight, current body weight and postnatal age using data from neonates receiving the simplified dosing regimen and the pharmacokinetic model of De Cock et al. (1). The red and blue lines in the plots represent the smoothed curves fitted to the data points.

**
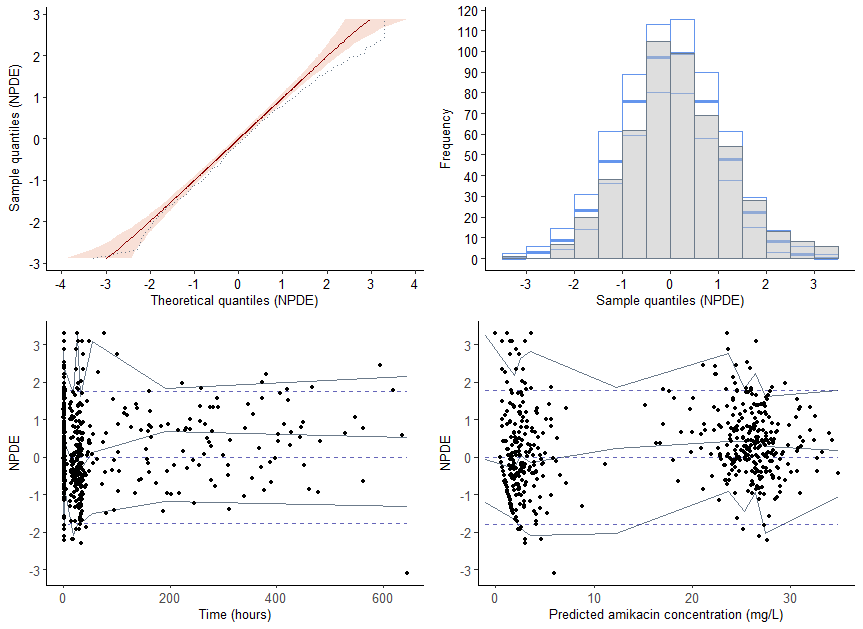
NPDE analysis**

**Figure S3:** Results obtained from the Normalized Prediction Distribution Error (NPDE) analysis, utilizing using data from neonates receiving the simplified dosing regimen and the pharmacokinetic model of De Cock et al. (1). The NPDE distribution displays a mean of 0.1763 and a variance 1.152. The mean is statistically significant different from 0 (p<0.05) as determined by the Wilcoxon signed-rank test, while the variance is not statistically significant different from 1 (p<0.05) with the Fisher test of variance.

**
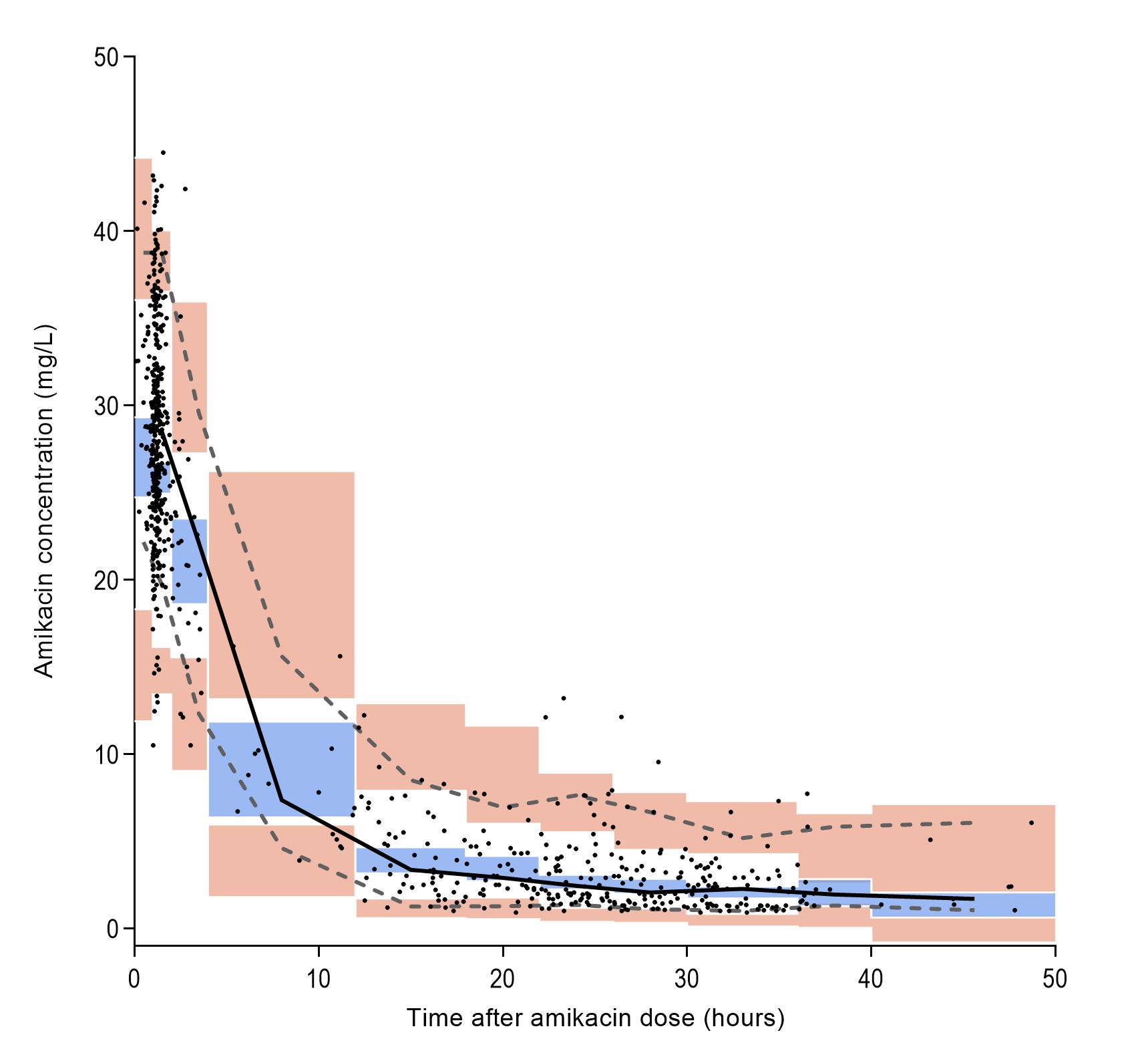
****VPC**

**Figure S4:** Visual predictive check (VPC) utilizing data from neonates receiving the simplified dosing regimen and the pharmacokinetic model of De Cock et al. (1). The closed circles represent the observed amikacin concentrations. The grey dotted lines represent the predicted percentiles. The blue area indicates the 95% confidence interval of the model-predicted median and the red area indicates the model-predicted 5th and 95th percentiles. The model generally captured the data reasonably since the observed medians of the 5th, 50th and 95th percentiles lay within the 95% confidence interval of the model-predicted percentiles.

**Parameter re-estimation**

**Table S3:** Final parameter estimates for the pharmacokinetic model of De Cock et al. and re-estimated using data from neonates receiving the simplified dosing regimen (Amsterdam UMC dataset) (1).

| **Parameter** | **Estimate (CV%)^a^** | |
| --- | --- | --- |
|  | **De Cock et al. (1)** | **Amsterdam UMC dataset** |
| CL_p_ in CL = CL_p_ × (bBW/median)^m^ × (1+ n × [PNA/median]) × o (ibuprofen) | 0.049 (2.21) | 0.058 (7.4) |
| m | 1.34 (2.04) | 1.30 (3.8) |
| n | 0.213 (9.81) | 0.149 (17.2) |
| o^b^ | 0.838 (3.88) | 1 |
| *V*_p_ in *V*_1_ = *V*_p_ × (cBW/median)^p^ | 0.833 (1.34) | 0.746 (1.6) |
| p | 0.919 (2.46) | 0.906 (2.7) |
| Q = r × CL | 0.415 (12.3) | 0.340 (8.1) |
| V_2_ = V_1_ | V_2_ = V_1_ | V_2_ = V_1_ |
| IIV on CL | 0.0899 (14.9) | 0.136 (13.8) |
| Proportional error | 0.0614 (8.2) | 0.0506 (9.4) |
| Additive error | 0.267 (27.2) | 0.309 (35.0) |

Abbreviations: CL_p_, clearance (L/h); *V*_p_, central volume of distribution (L); bBW, body weight at birth (g); cBW, current body weight (g); PNA, postnatal age (days); *Q* = intercompartmental clearance (L/h); *V*_1_, central volume of distribution (L); *V*_2_, peripheral volume of distribution (L).
^#^Median values for amikacin PK model: bBW = 1,750 g, PNA = 2 days, cBW = 1,760 g; ^b^Equals 1 given very low ibuprofen concurrent use.

**Calculation of bias & imprecision
Table S4:** Amikacin pharmacokinetic model of De Cock et al. prediction error using data from neonates receiving the simplified dosing regimen (1). Prediction error is assessed through bias, represented by mean prediction error (MPE) in mg/L, and precision, depicted by root mean square error (RMSE) in mg/L.

| **Population-predicted concentrations** | | | |
| --- | --- | --- | --- |
|  | **All concentrations (n=768)** | **C_min_ (n=242)** | **C_max_ (n=402)** |
| **MPE (mg/L)** | -0.44 (-0.78 to -0.10) | -0.04 (-0.28 to -0.21) | -2.13 (-2.88 to -1.74) |
| **RMSE (mg/L)** | 4.98 (1.47 to 8.49) | 1.96 (0.57 to 3.36) | 6.27 (0.48 to 12.06) |

| **Individual-predicted concentrations** | | | |
| --- | --- | --- | --- |
|  | **All concentrations (n=768)** | **C_min_ (n=242)** | **C_max_ (n=402)** |
| **MPE (mg/L)** | -0.27 (-0.58 to 0.03) | 0.01 (-0.10 to 0.11) | -2.14 (-2.68 to -1.61) |
| **RMSE (mg/L)** | 4.50 (1.40 to 7.60) | 0.84 (0.64 to 1.05) | 5.93 (0.73 to 11.13) |

**Supplementary data 4: Results of optimization of amikacin population PK model**

Re-estimation of the final PK parameters using the model-building dataset closely resembled those reported by De Cock et al. (1), except for the proportional error, which decreased with the inclusion of IIV on V1 (**Table S5**). The individual-predicted versus observed concentrations (**Figure S5**) and VPC (**Figure S7**) indicated accuracy in predicting C_min_ values consistent with the original model, and more precise prediction of C_max_ values upon incorporating IIV on V1. When applying the optimized PK model to the validation dataset, goodness-of-fit plots and VPC demonstrated a similar accurate prediction of both C_min_ and C_max_ values (**Figure S5 and S7**). IIV in clearance was evenly distributed for PNA, birth weight and current weight for the model-building dataset and external dataset (**Figure S6**).

To validate the appropriateness of a 30% IIV on V1 for our data, sensitivity analyses were conducted. Different IIV values for V1 ranging from 0 to 50% were integrated into the refitted PK model. The individual-predicted versus observed concentrations for the model-building dataset were visually inspected, alongside calculation of bias and imprecision for the simplified dosing dataset.

A noticeable improvement in the prediction of C_max_ values was observed up to an IIV of 30%, beyond which this improvement plateaued (**Figure S8**). Incorporating a 30% IIV on V1 resulted in a substantial decrease in the RMSE for C_max_ values to 2.8 mg/L (95%CI: 1.6 to 4.1 mg/L), and MPE to -1.0 mg/L (95%CI: -1.3 to -0.8 mg/L), which was deemed clinically acceptable.

**Parameter re-estimation with fixed interpatient variability (30%) on volume of distribution**

**Table S5:** Final parameter estimates for the pharmacokinetic model of De Cock et al. and re-estimated using the model-building dataset (=optimized PK model) (1).

| **Parameter** | **Estimate (CV%)** | |
| --- | --- | --- |
|  | **De Cock et al. ^a^  (1)** | **Model-building dataset (optimized PK model)^b^** |
| CL_p_ in CL = CL_p_ × (bBW/median)^m^ × (1+ n × [PNA/median]) × o (ibuprofen) | 0.049 (2.21) | 0.044 (3.2) |
| m | 1.34 (2.04) | 1.34 (2.4) |
| n | 0.213 (9.81) | 0.597 (7.8) |
| o^c^ | 0.838 (3.88) | 1 |
| *V*_p_ in *V*_1_ = *V*_p_ × (cBW/median)^p^ | 0.833 (1.34) | 0.794 (1.5) |
| p | 0.919 (2.46) | 0.903 (2.6) |
| Q = r × CL | 0.415 (12.3) | 0.468 (7.8) |
| V_2_ = V_1_ | V_2_ = V_1_ | V_2_ = V_1_ |
| IIV on CL | 0.0899 (14.9) | 0.0856 (11.3) |
| IIV on V_1_ |  | 0.09 |
| Proportional error | 0.0614 (8.2) | 0.0279 (13.7) |
| Additive error | 0.267 (27.2) | 0.378 (17.5) |

Abbreviations: CL_p_, clearance (L/h); *V*_p_, central volume of distribution (L); bBW, body weight at birth (g); cBW, current body weight (g); PNA, postnatal age (days); *Q* = intercompartmental clearance (L/h); *V*_1_, central volume of distribution (L); *V*_2_, peripheral volume of distribution (L).
^a^Median values for amikacin PK model De Cock: bBW = 1,750 g, PNA = 2 days, cBW = 1,760 g;
^b^Median values for combined dataset: bBW = 1,685 g, PNA = 5 days, cBW = 1,700 g;
^c^Equals 1 given low ibuprofen concurrent use.

**
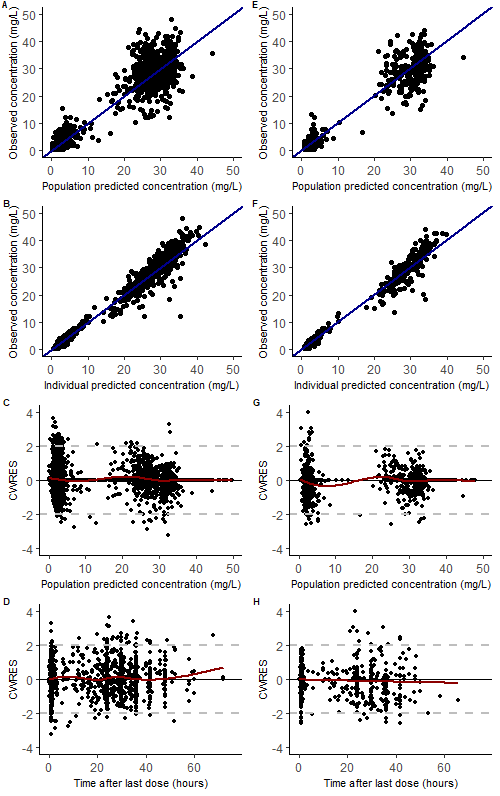
Goodness-of-fit plots optimized amikacin PK model**

**Figure S5:** Goodness-of-fit plots for the optimized pharmacokinetic model for the model-building dataset (A-D) and validation dataset (E-H). The blue line represents the line of unity and the red line represents the correlation of observations and predictions. CWRES, conditional weighted residuals.

**Interindividual variability on amikacin clearance and fixed on volume of distribution versus covariates**


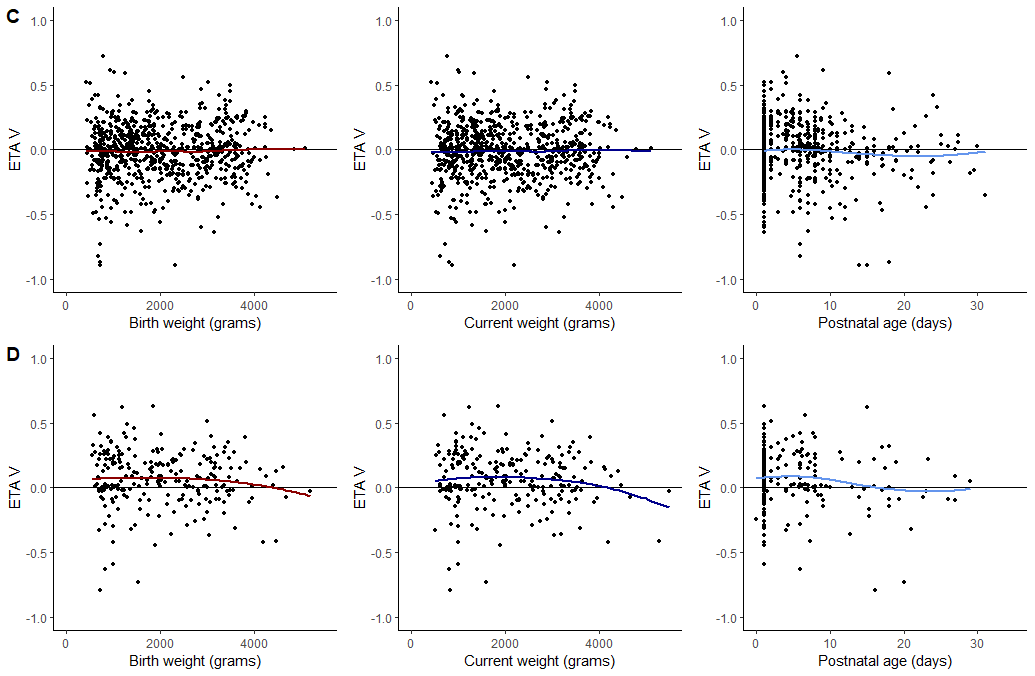

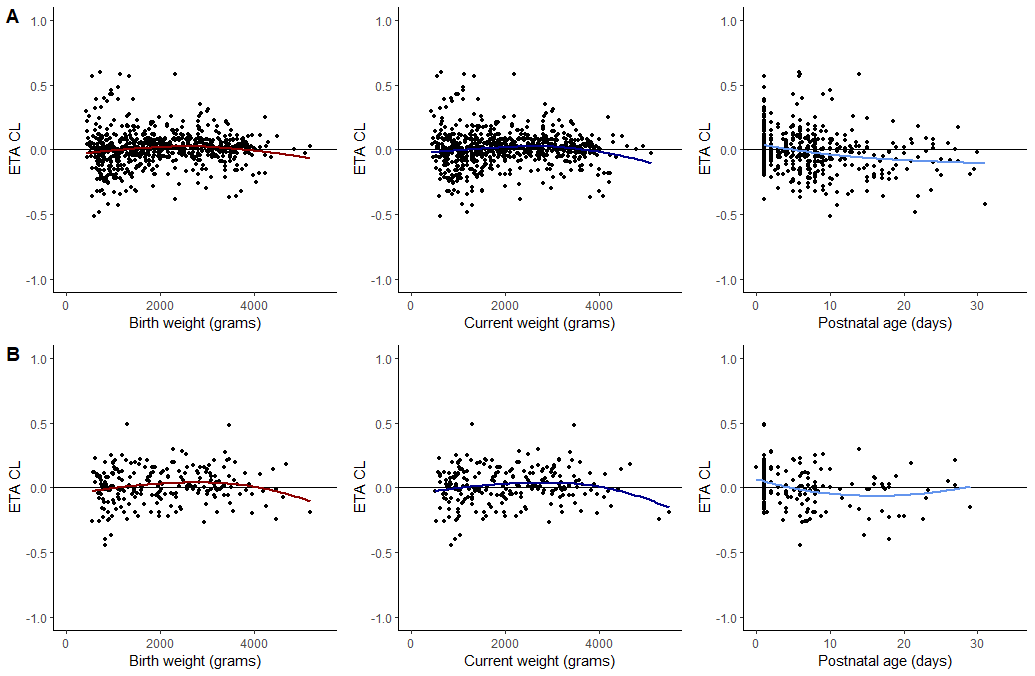


**Figure S6:** Interindividual variability (ETA) on clearance (CL) and central volume of distribution (V) versus birth weight, current weight and postnatal age for the optimized pharmacokinetic model using the model-building dataset (A, C) and the validation dataset (B, D).


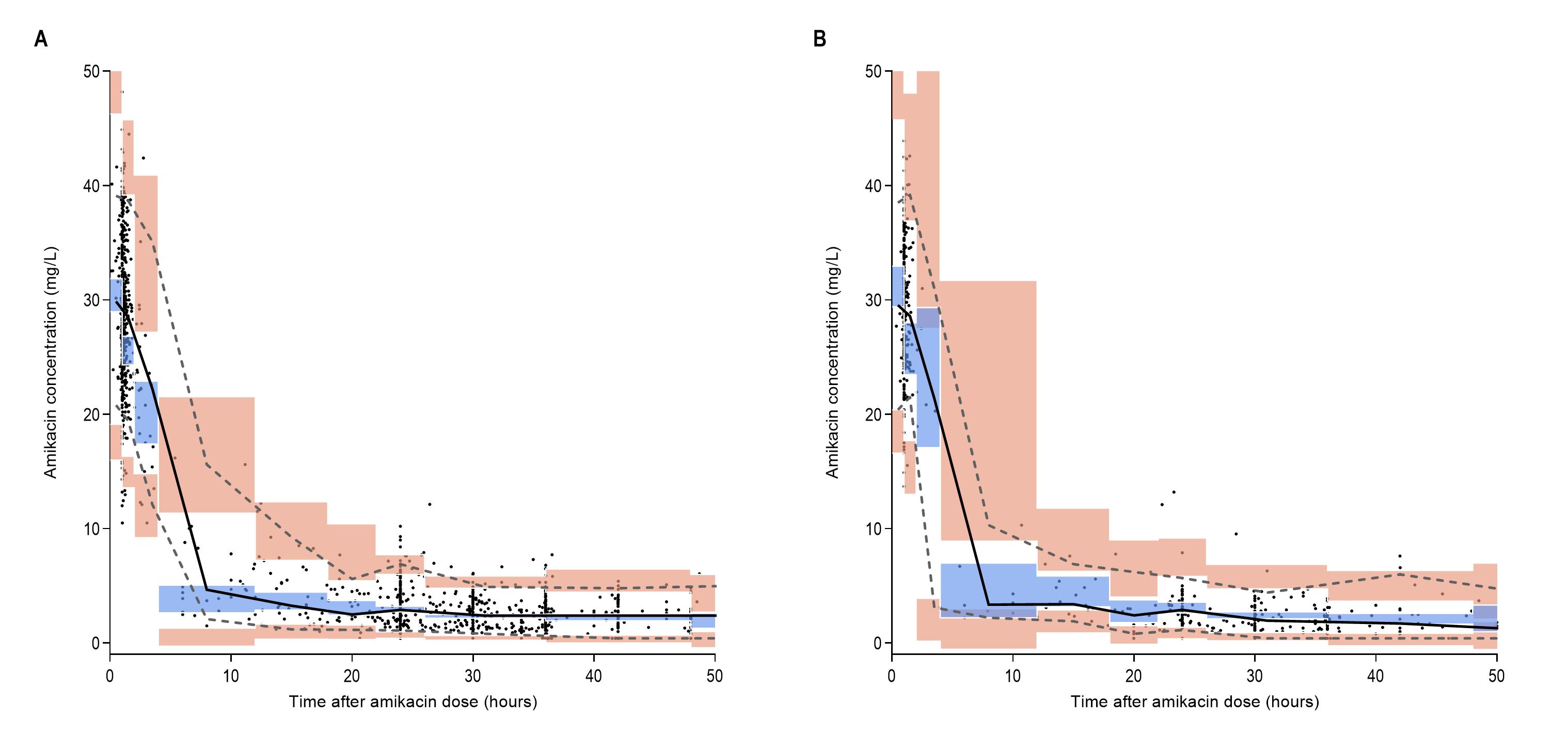
**VPC of optimized PK model (model-building and validation dataset)**

**Figure S7:** Visual predictive check (VPC) utilizing the **A)** model-building dataset or **B)** external dataset and the optimized pharmacokinetic model. The closed circles represent the observed amikacin concentrations. The grey dotted lines represent the predicted percentiles. The blue area indicates the 95% confidence interval of the model-predicted median and the red area indicates the model-predicted 5th and 95th percentiles. The model generally captured the data reasonably since the observed medians of the 5th, 50th and 95th percentiles lay within the 95% confidence interval of the model-predicted percentiles.

**
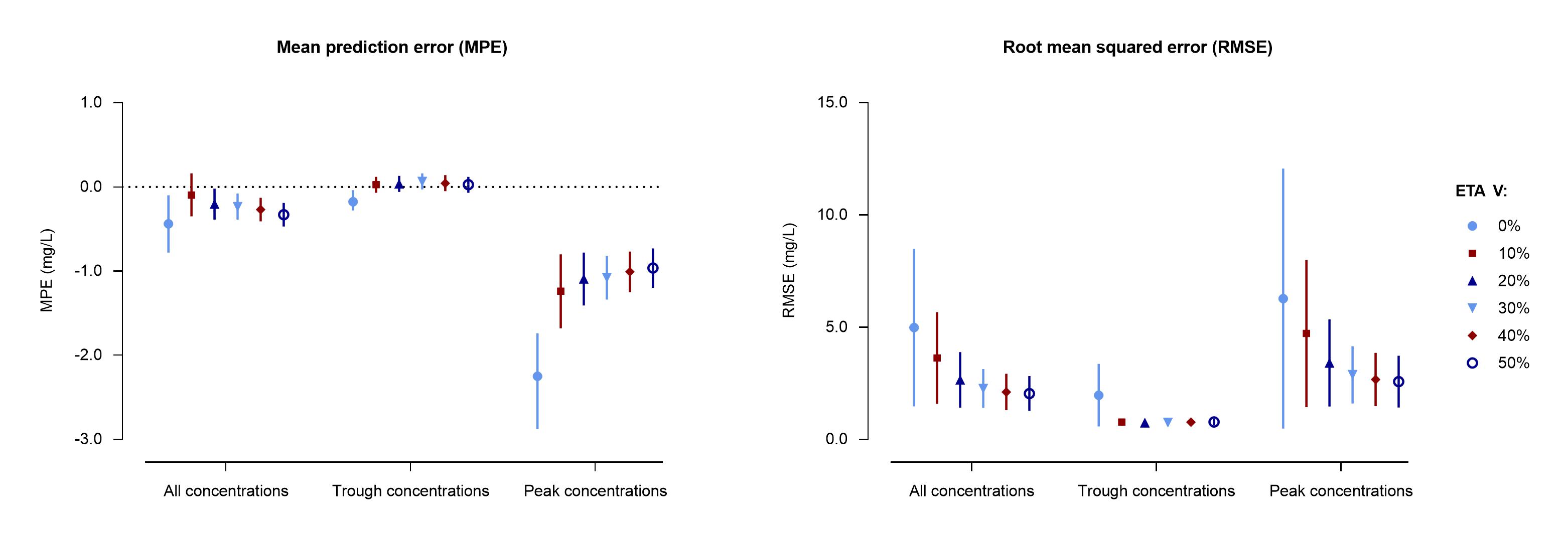
Calculation of bias & imprecision: refitted model with fixed interpatient variability on volume of distribution**

**Figure S8:** Amikacin refitted pharmacokinetic model using different fixed percentages of interpatient variability on central volume of distribution using data from neonates receiving the simplified dosing regimen. Prediction error was evaluated using the individual predicted amikacin concentrations (IPRED). Prediction error is assessed through bias, represented by Mean Prediction Error (MPE) in mg/L, and precision, depicted by Root Mean Square Error (RMSE) in mg/L.


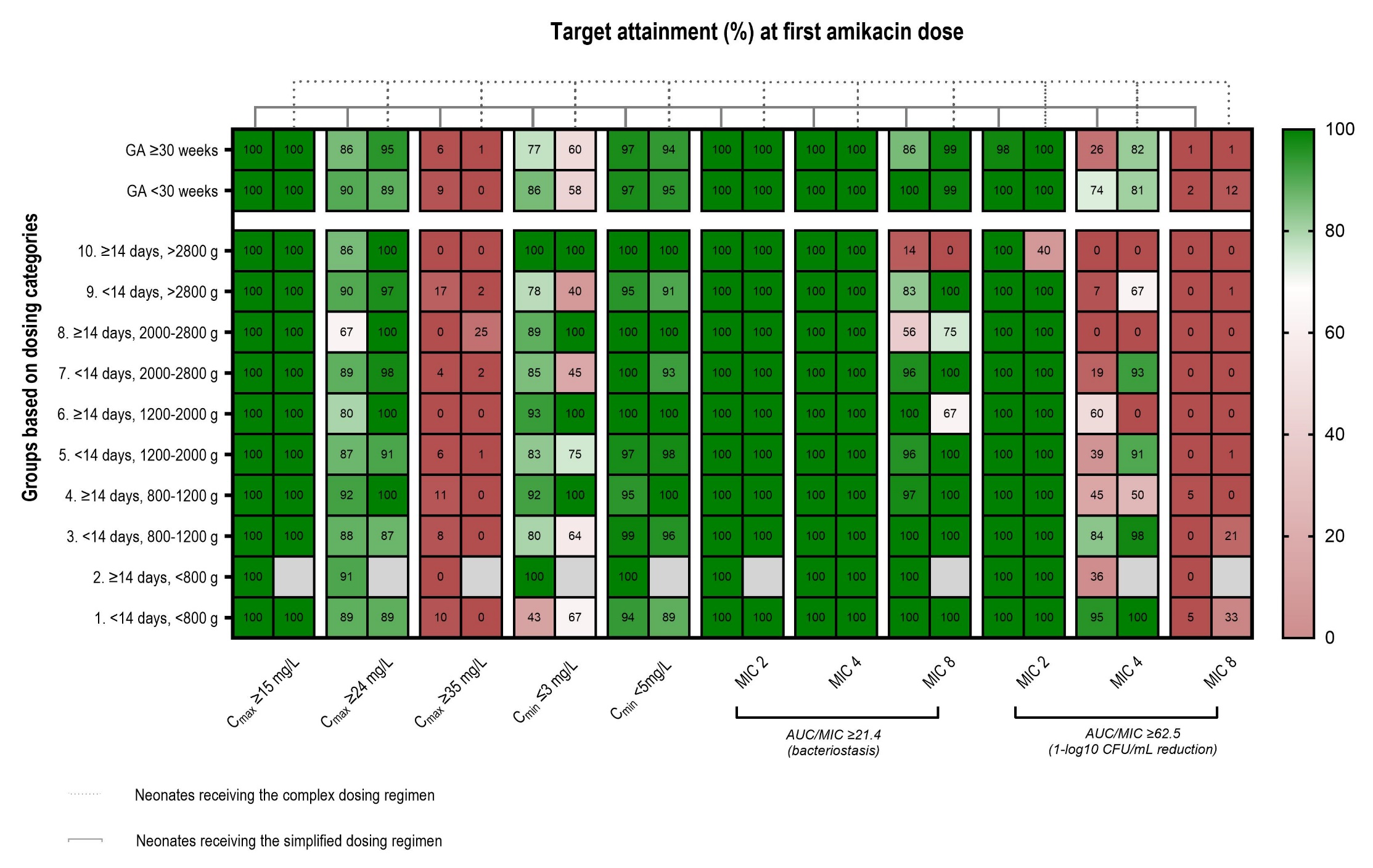
**Supplementary data 5: Results of target attainment per dosing subgroup**

**Figure S9:** Target attainment of amikacin for the first dose for neonates receiving the simplified dosing regimen or the complex dosing regimen per dosing category as defined for the simplified and complex dosing regimen. Target attainment is calculated for different targets for efficacy and toxicity, including peak concentrations (Cmax), area under the curve over minimum inhibitory concentration (AUC/MIC) for bacteriostasis or reduction, and trough concentrations (Cmin). The optimized pharmacokinetic model as described was used to calculate precise Cmin, Cmax and AUC values at the first dose.

**References**

1. De Cock RF, Allegaert K, Schreuder MF, Sherwin CM, de Hoog M, van den Anker JN, et al. Maturation of the glomerular filtration rate in neonates, as reflected by amikacin clearance. Clin Pharmacokinet. 2012;51(2):105-17.

2. Bergstrand M, Hooker AC, Wallin JE, Karlsson MO. Prediction-corrected visual predictive checks for diagnosing nonlinear mixed-effects models. AAPS J. 2011;13(2):143-51.

3. Brendel K, Comets E, Laffont C, Mentré F. Evaluation of different tests based on observations for external model evaluation of population analyses. J Pharmacokinet Pharmacodyn. 2010;37(1):49-65.

4. Bartelink IH, van Kesteren C, Boelens JJ, Egberts TC, Bierings MB, Cuvelier GD, et al. Predictive performance of a busulfan pharmacokinetic model in children and young adults. Ther Drug Monit. 2012;34(5):574-83.

5. Sheiner LB, Beal SL. Some suggestions for measuring predictive performance. J Pharmacokinet Biopharm. 1981;9(4):503-12.

6. Smits A, De Cock RF, Allegaert K, Vanhaesebrouck S, Danhof M, Knibbe CA. Prospective Evaluation of a Model-Based Dosing Regimen for Amikacin in Preterm and Term Neonates in Clinical Practice. Antimicrob Agents Chemother. 2015;59(10):6344-51.
